# Conservative Management of an Enterocutaneous Fistula Following Sigmoid Mesenteric Cyst Excision: A Case Report

**DOI:** 10.7759/cureus.95553

**Published:** 2025-10-28

**Authors:** Abhilash Mesh, Gayan Nanayakkara, Ahmed Aly, Mohammed Elmorsy

**Affiliations:** 1 Medicine, Withybush Hospital, Haverfordwest, GBR; 2 Surgery, Withybush Hospital, Haverfordwest, GBR; 3 General Surgery, Withybush Hospital, Haverfordwest, GBR

**Keywords:** enterocutaneous fistula, general surgery, pathology, radiology, sigmoid mesenteric cyst

## Abstract

We present the case of a 64-year-old woman who developed a postoperative small bowel-cutaneous enterocutaneous fistula (ECF) following excision of a large sigmoid mesenteric duplication cyst. Mesenteric duplication cysts are rare congenital anomalies, most frequently arising in the ileum, with sigmoid involvement being exceptionally uncommon in adults. They may present with abdominal pain, mass effect, or be discovered incidentally, and complete surgical excision is the treatment of choice. However, postoperative complications such as ECF formation are uncommon and can be challenging to manage.

In this case, the patient underwent elective resection of a large sigmoid mesenteric duplication cyst, which was densely adherent to adjacent small bowel loops and the anterior abdominal wall. The immediate postoperative period was complicated by persistent purulent wound discharge and localised abdominal wall erythema. Contrast-enhanced computed tomography demonstrated a fistulous tract originating from a segment of small bowel and communicating with the anterior abdominal wall skin. Microbiological cultures from wound swabs grew mixed enteric organisms, guiding targeted antibiotic therapy.

Given the patient’s hemodynamic stability, absence of diffuse peritonitis, and low-to-moderate fistula output, a conservative management approach was adopted. This included total parenteral nutrition to achieve bowel rest and correct nutritional deficiencies, regular wound care, and intravenous broad-spectrum antibiotics adjusted according to microbiology results. Over subsequent weeks, the fistula output progressively decreased, wound healing improved, and inflammatory markers normalised. Follow-up imaging demonstrated near-complete closure of the fistulous tract. Surgical re-intervention was avoided.

This case highlights that, in appropriately selected patients, conservative management of postoperative small bowel-cutaneous fistulas can lead to excellent outcomes. Factors favouring nonoperative treatment included the patient’s clinical stability, controlled sepsis, adequate nutritional support, and the absence of distal obstruction. The role of multidisciplinary care, involving surgeons, radiologists, microbiologists, and nutrition specialists, was critical in achieving a favourable result.

We also underscore the rarity of sigmoid mesenteric duplication cysts in the adult population and the importance of recognising fistula formation as a possible complication when the lesion is adherent to the small bowel and anterior abdominal wall. Individualised treatment planning remains essential, balancing the risks of surgery against the potential for spontaneous fistula closure under optimal conservative care.

## Introduction

Mesenteric duplication cysts are rare congenital anomalies of the gastrointestinal tract, characterised by cystic or tubular structures that share a common blood supply with adjacent bowel. They most frequently occur in the ileum, whereas sigmoid mesenteric duplication cysts represent an exceptionally uncommon presentation, particularly in adults [1,2]. Their exact aetiology remains uncertain, with proposed mechanisms including aberrant recanalisation of the primitive gut, partial twinning, and intrauterine vascular accidents [3,4].

Clinical presentation is variable and depends on the cyst’s size, location, and relationship to surrounding structures. While some lesions are discovered incidentally, others present with abdominal pain, a palpable mass, bowel obstruction, gastrointestinal bleeding, or, rarely, infection [5,6]. Surgical excision remains the mainstay of treatment, with the aim of complete resection to prevent recurrence and complications [7].

Although most patients recover uneventfully, complications may arise when cysts are densely adherent to the adjacent bowel or abdominal wall. One such rare complication is the development of an enterocutaneous fistula (ECF), an abnormal communication between the gastrointestinal tract and skin. ECFs pose significant management challenges due to risks of sepsis, electrolyte imbalance, malnutrition, and prolonged hospitalisation [8-10]. Radiographic and endoscopic studies are important for defining fistula anatomy and guiding therapy [9,11]. In selected cases, interventional radiology may assist with drainage of abscess-fistula complexes or percutaneous catheter management [6,7].

Management strategies range from early surgical intervention to conservative approaches such as nutritional optimisation, infection control, and wound care. The choice is dictated by fistula anatomy, output, and patient stability [1,8,10]. While many fistulas close with conservative management, persistence is common in Crohn’s disease, and some cases ultimately require resection [2,3]. Iatrogenic causes, including colonoscopic perforation, have also been reported as precipitants of complex fistula formation [4,5].

We report the case of a 64-year-old woman who developed a postoperative small bowel-cutaneous fistula following excision of a large sigmoid mesenteric duplication cyst. This case is notable for the rarity of the primary pathology, the unusual postoperative complication, and the successful outcome achieved with conservative management using total parenteral nutrition (TPN) and multidisciplinary support. It underscores the importance of individualised treatment strategies in rare surgical complications.

## Case presentation

A 64-year-old woman was referred to the Gynaecology service with a suspected pelvic mass. She reported increasing abdominal distension and discomfort over several months but denied any acute abdominal pain, change in bowel habit, weight loss, or constitutional symptoms. There was no history of abnormal vaginal bleeding.

Her past surgical history was significant for a total abdominal hysterectomy and bilateral salpingo-oophorectomy (TAH + BSO) performed in 2010 for benign gynaecological disease, as well as a previous appendicectomy. Operative records from her earlier surgeries documented the presence of extensive intra-abdominal adhesions, which had complicated both procedures. She had no significant medical comorbidities and was not on long-term medication. Family history was unremarkable, and she was a lifelong non-smoker.

On clinical examination, she was well and hemodynamically stable. Abdominal palpation revealed a firm, immobile mass extending from the pelvis up to just below the xiphisternum. The mass was predominantly cystic in nature. There was no associated tenderness or clinical evidence of ascites.

**Figure 1:**
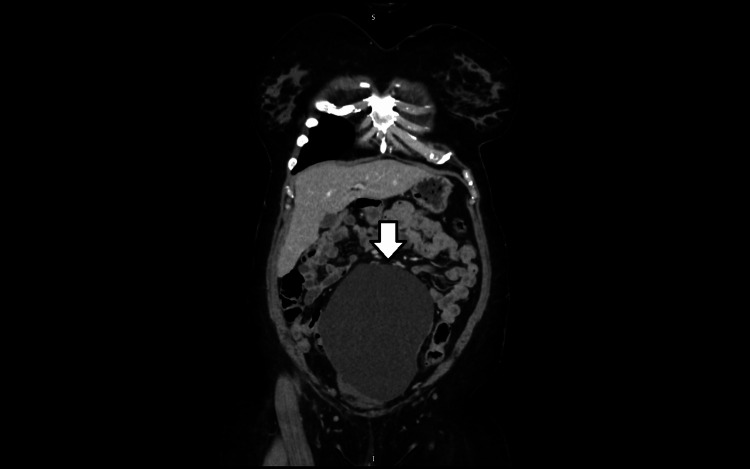
Coronal CT image showing a well-defined cystic lesion in the lower abdomen and pelvis. The lesion demonstrates homogeneous low attenuation without internal septations or solid components. Cross-sectional imaging demonstrated a large cystic lesion measuring approximately 19 cm in maximum dimension

**Figure 2:**
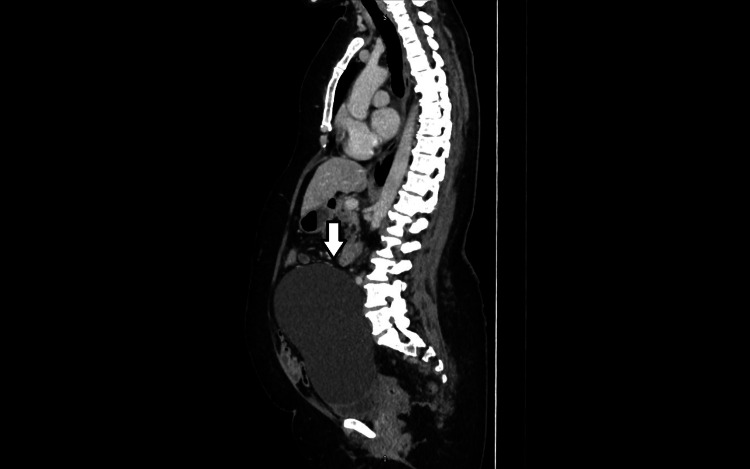
Sagittal CT image of the same cystic lesion adjacent to the sigmoid colon, which was confirmed intra-operatively to arise from the sigmoid mesentery. Cross-sectional imaging demonstrated a large cystic lesion measuring approximately 19 cm in maximum dimension

Preoperative investigations included routine blood tests, which were within normal limits. Tumour marker CA-125 was not elevated. Cross-sectional imaging demonstrated a large cystic lesion measuring approximately 19 cm in maximum dimension, raising the differential diagnoses of an ovarian remnant cyst versus a mesenteric cyst. Given her history of hysterectomy and oophorectomy, a non-ovarian origin was suspected but could not be confirmed radiologically (Figures 1, 2).

The case was reviewed at the Gynaecology Multidisciplinary Team (MDT) meeting. After discussion, the consensus was to proceed with exploratory laparotomy under general anaesthesia, with input from both Gynaecology and General Surgery due to the complexity of the patient’s surgical history and the uncertainty regarding the origin of the cyst.

Intraoperative findings

At examination under anaesthesia (EUA), a firm, immobile cystic mass was again palpated in the lower abdomen. A midline sub-umbilical incision was performed with careful entry into the peritoneal cavity due to anticipated adhesions from previous surgeries. Intraoperatively, extensive fibrotic adhesions involving both small and large bowel were encountered, particularly beneath the rectus sheath and within the pelvis, which was densely adherent and partially inaccessible. During mobilisation, the cyst was identified as arising from the mesentery of the sigmoid colon. Approximately two litres of clear cystic fluid were aspirated and sent for urgent cytological and histopathological assessment. The cyst wall was meticulously dissected and excised in its entirety under the supervision of the surgical consultant, who subsequently assumed operative management. No visible bowel injury or enterotomy was identified intra-operatively. The total operative time was approximately two hours, and haemostasis was secured before closure.

The procedure was uneventful, and histopathology confirmed a benign cyst. The cyst fluid was also sent for CBNAAT, bacterial culture and sensitivity, and fungal culture, all of which were negative for any infectious disease. The patient had a smooth initial recovery: by postoperative day (POD) 3, she was tolerating a normal diet, had normal bowel function, and was discharged home.

During the first postoperative week, she remained well. However, on POD 9, she re-presented with abdominal pain and increasing tenderness around her lower midline wound. The wound, approximately 20 cm long and closed with clips, was inflamed and tender. Blood tests (Table 1) demonstrated clear evidence of a severe systemic inflammatory response, with marked leucocytosis and a significantly elevated inflammatory marker. Liver function tests showed a mixed pattern of hepatocellular and cholestatic derangement, raising concern for biliary tract involvement. Renal function was mildly impaired, although serum electrolytes remained within normal limits. Haematological indices, including haemoglobin concentration and platelet count, were preserved.

**Table 1:** Blood Tests on Re-Admission WBC: White Blood Cell; Hb: Haemoglobin; PLT: Platelets; RBC: Red Blood Cell; Hct: Haematocrit; MCV: Mean Cell Volume; MCH: Mean Cell Haemoglobin; RDW: Red Cell Distribution Width; CRP: C-reactive Protein; ALP: Alkaline Phosphatase; ALT: Alanine Transaminase; eGFR: Estimated Glomerular Filtration Rate; g/L: grams per litre; pg: picograms; fL: femtolitres; U/L: units per litre; mg/L: milligrams per litre; mmol/L: millimoles per litre; µmol/L: micromoles per litre; ×10⁹/L: billions per litre; ×10¹²/L: trillions per litre; mL/min/1.73 m²: millilitres per minute per 1.73 square metres of body surface area

Test	Result	Units	Reference Range
White blood cell (WBC)	20.5	x10^9/L	4.0 - 11.0
Neutrophils	18.3	x10^9/L	1.7 - 7.5
Lymphocytes	0.9	x10^9/L	1.0 - 4.5
Monocytes	1	x10^9/L	0.2 - 0.8
Haemoglobin	128	g/L	115 - 165
Platelets	296	x10^9/L	150 - 400
Red blood cells (RBC)	4.49	x10^12/L	3.8 - 5.5
Haematocrit	0.4	L/L	0.37 - 0.47
C-reactive protein (CRP)	350	mg/L	< 5
Alanine transaminase (ALT)	122	U/L	< 33
Alkaline phosphatase (ALP)	410	U/L	30 - 130
Bilirubin	20	µmol/L	< 21
Albumin	41	g/L	35 - 50
Creatinine	97	µmol/L	46 - 92
eGFR	50	mL/min/1.73m²	≥ 60
Urea	6.8	mmol/L	2.5 - 7.8
Sodium	139	mmol/L	133 - 146
Potassium	4.2	mmol/L	3.5 - 5.3
Random glucose	7.3	mmol/L	3.0 - 7.7

**Figure 3:**
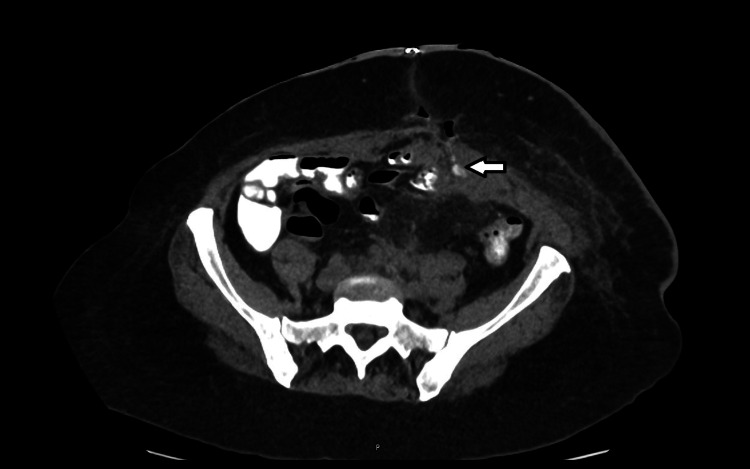
Axial view with an enterocutaneous fistula (white arrow) Repeat CT scan showing the fistula

The wound was reopened, releasing haemoserous fluid. Over the next few days, the nature of the discharge changed. By POD 12, the initially purulent drainage had turned yellow and faeculent, consistent with the development of an ECF. At this point, the wound was producing around 50-75 ml of bilious fluid daily. A repeat CT scan confirmed persistent collection and bile-stained fistula output (Figure 3).

Microbiology recommended targeted antibiotic therapy, while nutritional support was initiated with TPN under the guidance of the dietetics team.

Despite this complication, the patient remained afebrile, haemodynamically stable, and showed no signs of peritonitis or necrotising fasciitis. The wound bed was healthy and granulating, with the anterior rectus sheath intact. The midline incision was left open for drainage and extended by 10 cm to facilitate further evacuation.

The fistula led to persistent enteric leakage, necessitating meticulous wound care and ongoing nutritional support. The patient was kept nil per mouth (NPO) to allow bowel rest, and TPN was maintained for the majority of her admission, providing essential caloric and protein requirements until bowel function improved.

In the immediate postoperative period (Week 1), the patient was managed with intravenous broad-spectrum antibiotics, wound care, and careful monitoring of fluid and electrolyte balance. The abdominal wound showed mild serous discharge, and bowel sounds gradually returned by the end of the first week.

During Week 2, the wound output increased and was noted to contain enteric content, confirming the presence of an ECF. The patient was maintained on intravenous antibiotics and commenced on TPN to reduce fistula output and support nutritional status.

By Week 3, the fistula output began to decrease, and the surrounding skin condition improved with regular wound care and protective dressings. The patient remained afebrile and clinically stable throughout this period.

In Week 4, antibiotic therapy was transitioned to oral form, and gradual reintroduction of enteral feeding was initiated. The wound continued to contract, and output further diminished.

By Week 5, the fistula output had nearly ceased, with evidence of spontaneous closure and healthy granulation tissue formation. Nutritional intake was well tolerated, and electrolyte levels remained stable.

By Week 6, the wound had healed significantly, and the patient was tolerating a full enteral diet with normal bowel function. She was subsequently discharged with outpatient follow-up.

At the outpatient 6-month follow-up, she demonstrated excellent recovery. The abdominal wound had almost completely healed, with only a small superficial opening discharging a minor amount of serous fluid, which required no further intervention. Bowel function had normalised, her appetite had returned, and there was no evidence of infection, malnutrition, or recurrent fistula formation.

## Discussion

ECFs are among the most demanding postoperative complications, driving morbidity through fluid/electrolyte derangements, protein-calorie malnutrition, sepsis risk, and prolonged hospitalisation [1,2]. Contemporary management follows the “SNAP” principles: Sepsis control, Nutrition optimisation, delineation of Anatomy, and formulation of an operative Plan, applied by a coordinated multidisciplinary team (surgery, microbiology, dietetics, wound/ostomy nursing, radiology, and interventional radiology) [1,3].

Rationale for conservative care in stable patients

In the absence of uncontrolled sepsis, distal obstruction, or hostile abdomen, initial non-operative management is preferred because a meaningful proportion of postoperative ECFs will close spontaneously once infection is controlled and nutrition restored [1,3]. Predictors of spontaneous closure include low output, longer tract length, absence of distal obstruction, good nutritional status, and no ongoing inflammation or foreign body; adverse factors include high output, short tract, epithelialised tract, active sepsis, radiation or malignancy, and steroid use [1,3]. Our patient’s haemodynamic stability, lack of peritonitis, healthy granulating wound, and progressive reduction in output created favourable conditions for a conservative pathway, leading to spontaneous closure without reoperation.

Nutrition as therapy, not merely support

Malnutrition perpetuates fistula output and impairs wound healing; aggressive nutritional repletion is therefore therapeutic [1,3]. Early initiation of TPN can reduce catabolism, maintain nitrogen balance, and support immune function while the gut is not usable, with a planned transition to enteral nutrition as output falls and bowel function returnsexactly the trajectory in this case [1,3]. This approach aligns with large institutional experiences in ECF care that link meticulous nutrition with higher closure rates and lower mortality [2].

Sepsis and source control

Timely control of sepsis, through culture-guided antibiotics and drainage of abscess-fistula complexes, is the cornerstone of early management [1]. Image-guided percutaneous drainage and catheter-based strategies can downstage complex collections, protect the skin, and sometimes reduce fistula output, avoiding premature laparotomy in a hostile field [4,5]. Our patient required only targeted antibiotics and local wound strategies, with no drainable collection identified.

Define anatomy, then decide on timing

Cross-sectional imaging (contrast CT; MR enterography where appropriate) and selective fistulography/endoscopy clarify the fistula’s course, output drivers, and downstream patency, informing decisions about safe refeeding and any delayed operative plan [6,7]. In broadly accepted algorithms, definitive surgery, if needed, is deferred until inflammation has settled, nutritional indices normalise, and the abdomen is more amenable, typically several months from index surgery [1,3]. This delay reduces operative difficulty, recurrence, and short-bowel risk.

Several published reports have described successful conservative management of postoperative ECFs, particularly in clinically stable patients without uncontrolled sepsis or distal obstruction. In these cases, multidisciplinary care focusing on nutritional optimisation, sepsis control, wound management, and electrolyte balance has been shown to promote spontaneous closure in up to 70-80% of patients within six to eight weeks [8,10]. Similar to our case, non-operative management was favoured when the patient remained haemodynamically stable and demonstrated a gradual reduction in fistula output. Early initiation of nutritional support, including parenteral or enteral routes as tolerated, and meticulous wound care were key contributors to recovery. While Crohn’s- and trauma-related fistulae often require disease-specific interventions, postoperative cases such as ours highlight the effectiveness of a stepwise conservative approach when anatomical and physiological conditions permit.

What this case adds

This report reinforces that in clinically stable postoperative ECFs, a conservative, protocolised strategy, sepsis control, skin/wound protection, strict fluid/electrolyte management, and nutrition first, can achieve closure without the hazards of early reoperation. The patient’s course (afebrile, stable physiology, falling output, progressive granulation, successful TPN then re-establishment of enteral nutrition) exemplifies best practice and supports guideline-concordant avoidance of premature surgical intervention [1,3,6].

Clinical takeaway

For postoperative ECFs without red flags (uncontrolled sepsis, distal obstruction, ischemic bowel, or abdominal catastrophe), conservative management should be the default initial strategy. It reduces operative morbidity, allows fistula maturation or closure, and creates safer conditions should delayed reconstruction be required [1,3,6].

## Conclusions

This case highlights the rarity of sigmoid mesenteric duplication cysts and the potential for complex postoperative complications such as ECFs. Although the patient initially presented with sepsis, prompt identification, targeted antibiotic therapy, and supportive management led to clinical stabilisation. Subsequent conservative treatment, comprising TPN, infection control, and multidisciplinary care, resulted in complete recovery without the need for surgical re-intervention. This outcome underscores the importance of individualised, non-operative management in selected cases, once sepsis is controlled, as re-operation in a hostile abdomen may carry substantial risk. Conservative therapy can therefore remain a valuable strategy in the management of postoperative enterocutaneous fistulae, offering the potential for spontaneous closure and favourable outcomes without additional operative morbidity.
